# Case report: Right atrial appendage hybrid access to bailout a stuck stent from the inferior vena cava of a small child

**DOI:** 10.3389/fcvm.2022.1084170

**Published:** 2023-01-25

**Authors:** Radityo Prakoso, Aditya Agita Sembiring, Latifa Hernisa, Brian Mendel, Olfi Lelya, Oktavia Lilyasari

**Affiliations:** ^1^Department of Cardiology and Vascular Medicine, Division of Pediatric Cardiology and Congenital Heart Disease, National Cardiovascular Center Harapan Kita, Universitas Indonesia, Jakarta, Indonesia; ^2^Division of Pediatric and Congenital Heart Surgery, National Cardiovascular Centre of Harapan Kita, Universitas Indonesia, Jakarta, Indonesia; ^3^Department of Cardiology and Vascular Medicine, Sultan Sulaiman Government Hospital, Serdang Bedagai, Sei Rampah, Indonesia

**Keywords:** ductal stenting, inferior vena cava, patent ductus arteriosus, single ventricle, strutted stent

## Abstract

A three-month-old baby boy (5. 4 Kg) with pulmonary atresia, subaortic ventricular septal defect (VSD), and patent ductus arteriosus (PDA) was sent for ductal stenting from the femoral vein. The route to the PDA was extremely tortuous and the procedure was complicated with a stent stuck in the abdominal inferior vena cava (IVC). Transfemoral stent recapture was technically laborious and the stent was successfully recaptured across a 10-Fr right atrial appendage (RAA) hybrid access avoiding a cardiopulmonary bypass (CBP). The PDA was subsequently stented for the femoral artery with satisfactory clinical outcomes.

## Introduction

The integrity and configuration of the stents can be easily disrupted with harsh manipulations making percutaneous interventions difficult and technically more complex. Strutted stents within the vessels are challenging scenarios for interventional cardiologists and can be associated with serious adverse events such as embolism, disintegration of the surrounding tissue, and vascular trauma (1, 2). There are currently no protocols for recapturing embolized strutted stents using percutaneous methods. Herein, we report and describe the use of the right atrial appendage (RAA) hybrid access as a bailout to safely remove a strutted stent from the abdominal inferior vena cava (IVC) of a small three-month-old baby boy.

## Case illustration

**Figure 1:**
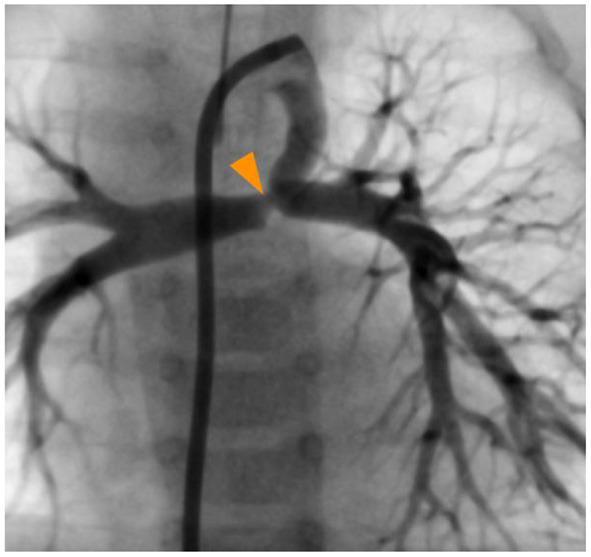
Ductal angiography showing the restricted pulmonary ductal (orange arrowhead) end and the absence of connection between the branch pulmonary arteries and the pulmonary artery trunk.

**Figure 2:**
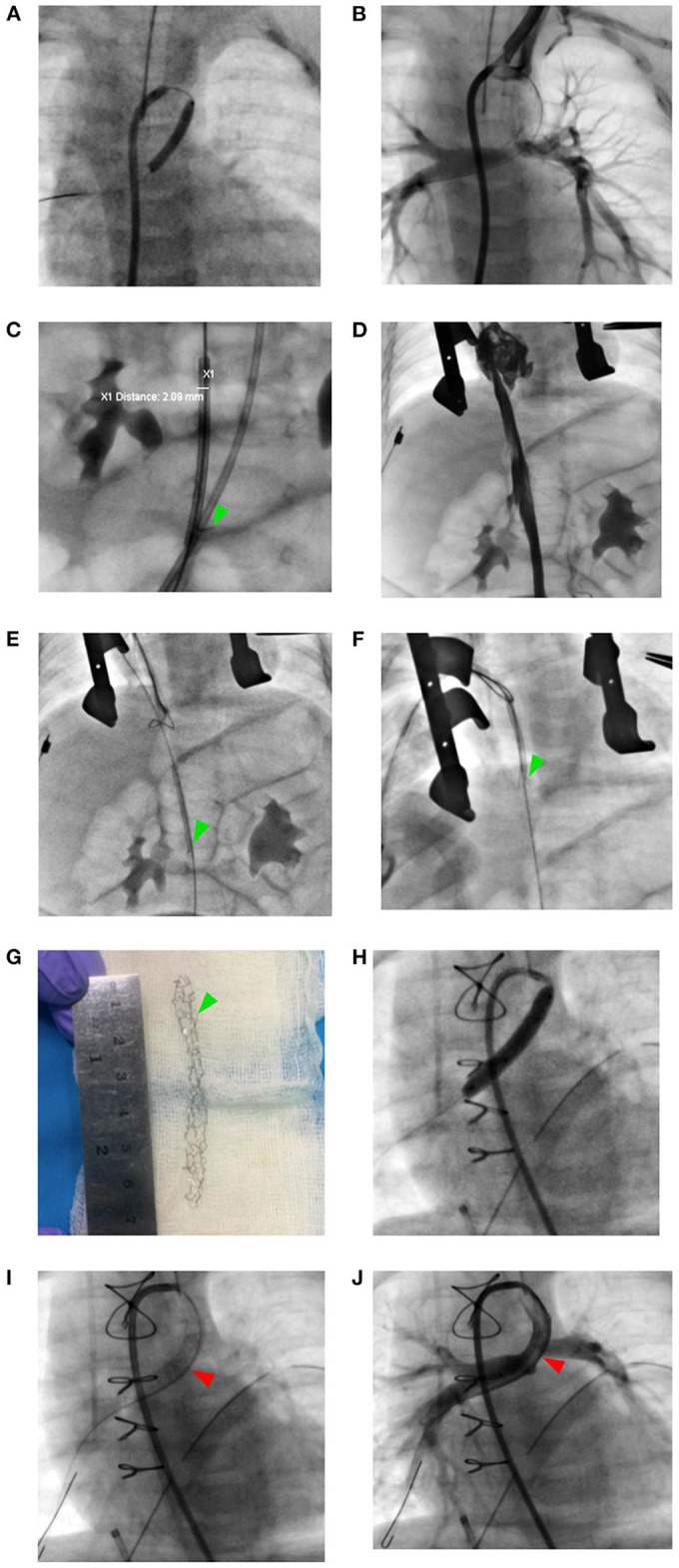
Stucked stent retrival strategy from the inferior vena cava and transarterial restenting the ductus arteriosus. **(A)** Ductal pre-dilation with an Ikazuchi coronary balloon 3.0 x 20 mm. **(B)** Failure to deliver the Dynamic vascular stent within the ductus arteriosus. **(C)** The stent strutted in the inferior vena cava (green arrowhead). **(D)** Caval angiography showing no vascular trauma. **(E–G)** Stent Evacuated through right atrial appendage hybrid access with 20 mm gooseneck snare. **(H, I)** Transarterial ductal restenting with Resolute Integrity 4.0 x 30 mm stent (red arrowhead). **(J)** Post-stenting ductal angiography.

A three-month-old boy (5.4 Kg) with severe cyanosis and diagnosed with type-II pulmonary atresia, subaortic 6.5 mm-large ventricular septal defect (VSD), and patent ductus arteriosus (PDA) with the saturation of only 40% was sent for femoral transvenous ductal stenting (DS). The case was discussed in a multidisciplinary meeting and DS was found more reasonable than surgical valvulotomy (Figure 1). From the right femoral vein, a 4-Fr 3.5 Judkins Right catheter was cannulated up to the PDA across the VSD. The PDA was pre-dilated with a 3.0 x 20 mm coronary balloon that was inflated to 6 atm (Figure 2A). A 0.035″ soft exchange wire was then positioned in the right pulmonary artery and a (6.0 x 38 mm) Dynamic vascular stent was delivered into position using the naked technique. It was technically impossible to push the stent inside the PDA because of the complex angulation (Figure 2B). The stent got stuck in the IVC upon retrieval (Figure 2C). We tried to push and pull the stent to verify that the stent is still on its wire track. Caval angiography was done to verify that the IVC was not damaged (Figure 2D). An urgent multidisciplinary decision was taken to remove the stent in the cath lab using a 10-Fr RAA hybrid access without cardiopulmonary bypass (CPB) support. The recapture of the stent was uneventfully performed using a 20 mm gooseneck snare (Figures 2E–G) and DS was successfully performed from the femoral artery with a (4.0 x 30 mm) Resolute Integrity stent that was inflated at 20 atm for 6 s (Figure 2H). Control angiography showed that the stent migrated toward the distal right pulmonary artery (Figures 2H, I). We decided to position a second stent because the PDA was not entirely covered. However, due to the complex PDA anatomy, it was technically difficult to overlap the first stent. We decided to abort our procedure since the PDA did not close at 3 months of age and the oxygen saturation had already risen to 94% (see Figure 2J). There were no vascular access complications. The follow-up was clinically satisfactory and the patient underwent Rastelli surgery age at 11 months with good outcomes.

## Discussions

### Ductal stenting in critical congenital heart disease

The DS procedure is an essential cornerstone in the interventional treatment of newborns with pulmonary flow duct-dependent circulation (3). A 0.014 coronary wire with a radio-opaque tip was used to determine the size of the stent where the length of the lesion was compared to the wire's tip. In this small patient, we chose the naked method and the more complex transvenous approach to first implant a larger stent and second avoid the potential injuries on the femoral artery and acute limb ischemia (4). At the time of the intervention, we had no prior experience with the more direct approach from the carotid or axillary arteries through which a 5 or even a 6-Fr sheath can be safely used even in small kids (5). As the distal part of the PDA was constricted, we also performed balloon dilatation to assure the safety of the stent implantation (6).

### Strutted stents in the abdominal inferior vena cava: Troubleshooting tactics

When we tried to retrieve the stent, the stent got stuck and strutted in the IVC. The stiffness of the Dynamic Vascular Stent and the rough maneuvering in the complex ductal loop may be to blame. Previous studies showed that coronary stent implantation in proximal segments of coronary arteries rather than distal ones may lead to dislodgement (7, 8). Its incidence ranges from 0.3 to 8% due to pre-mounting technologies and modern equipment (9). A limited number of reports described emergency surgical stent retrieval for entrapped coronary stents (8, 10). The majority of these cases are brought on by a balloon-stripped undeployed stent. This mechanism usually occurs due to constricted PDA, angulated lesions, short small stents, unexpanded stents, and manual handling of stents (11). The retrieving methods can be performed percutaneously, surgically, or a combination of both, which we used in this case. Percutaneous retrieval methods should be preferred if the patient's vital signs and clinical status are stable. Several retrieval methods are defined, including biliary forceps, twisted guide wires, multipurpose baskets, snares, and the small-balloon technique (12).

Extracting the stent through the RAA is a safe hybrid alternative but can be considered logistically challenging and there is a possibility of grazing the tricuspid valve, coronary sinus, and other surrounding structures (13). In this case, we tried to retrieve the stent by using a 90-gooseneck snaring system across an RAA hybrid access (13, 14). We decided to snare the stent through RAA access because transfemoral snaring would have certainly damaged the IVC or the femoral vein. Open abdominal surgery through the IVC was considered risky as air embolism can be detrimental in this case with an intracardiac shunt. Due to the IVC profundal location, surgical access to the clamp is also considered anatomically impossible with bad exposure to form purse-string sutures. Finally, the stent could be compressed and clamped when it is within the RAA, and damaged parts of the RAA could be surgically removed.

## Conclusions

Strutted stent entrapment is a rare but serious complication of transcatheter stenting interventions. Hybrid access across the RAA can be a safe and effective bailout solution to retrieve a trapped stent from the IVC without cardiopulmonary bypass support.

## Data availability statement

The original contributions presented in the study are included in the article/supplementary material, further inquiries can be directed to the corresponding author.

## Ethics statement

Ethical review and approval was not required for the study on human participants in accordance with the local legislation and institutional requirements. Written informed consent to participate in this study was provided by the participants' legal guardian/next of kin. Written informed consent was obtained from the minor(s)' legal guardian/next of kin for the publication of any potentially identifiable images or data included in this article.

## Author contributions

RP conceived the original idea of the manuscript. RP, AS, LH, and BM contributed in collecting the patient data and writing the main text of the paper. All authors discussed and agreed with the idea of the paper. The manuscript was proofread and accepted by all authors.
